# The implications of intensive care unit capacity strain for the care of critically ill patients

**DOI:** 10.5935/0103-507X.20160069

**Published:** 2016

**Authors:** Rachel Kohn, Scott D. Halpern, Meeta Prasad Kerlin

**Affiliations:** 1 Department of Medicine, Perelman School of Medicine, University of Pennsylvania - Philadelphia, Pennsylvania.; 2 Center for Clinical Epidemiology and Biostatistics, Perelman School of Medicine, University of Pennsylvania - Philadelphia, Pennsylvania.; 3 Leonard Davis Institute of Health Economics, Perelman School of Medicine, University of Pennsylvania - Philadelphia, Pennsylvania.

## Introduction

Every intensive care unit (ICU) has an inherent "capacity" or "ability to provide high-quality care for everyone who is or could become a patient in that ICU on a given day".^(1)^ As with any operation, an ICU's capacity is not without bounds. ICU capacity has been likened to a balloon - able to stretch to a point to accommodate more patients or higher acuity, but when capacity is exceeded, the balloon pops or care deteriorates.^(1)^ However, it is also possible that ICUs operate like motors - rather than exhibiting markedly different performance at an inflection point of demand, their efficiency may change as a continuous function of the demands placed on them. In this perspective, we discuss the evidence regarding what contributes to ICU capacity strain, identify key knowledge gaps in the field, and consider the implications for future research and patient care.

## What contributes to intensive care unit capacity strain?

In operations terms, capacity strain can be defined as "limited capacity and the resulting problems of waiting times and throughput losses".^(2)^ Strain may be caused by anything that results in a demand for resources in excess of those that are available. In health care settings, strain may result from sheer volume of patients. Indeed, this simple model of strain was first described in the Emergency Department (ED). Multiple studies have demonstrated that high patient volume ("crowding") in EDs is associated with adverse outcomes, including prolonged time to thrombolysis for acute myocardial infarctions^(3)^ and delayed or missed antibiotic administration for community-acquired pneumonia.^(4)^ A more recent study even demonstrated increased 90-day mortality in the setting of ED crowding,^(5)^ highlighting the potential importance of capacity strain not only on immediate processes of care but additionally on downstream patient outcomes.

Our research team has extended the concept of capacity strain to the ICU, and expanded upon its scope. We have shown that several factors contribute to the strain perceived by frontline clinicians in the ICU, including not only the number of patients, but also their severity of illness, the number of new admissions to the ICU, and even factors external to the ICU, such as the capacity of general wards to accept patients ready for ICU discharge.^(6)^

## What are the implications of intensive care unit capacity strain?

ICU capacity strain has far-reaching implications for ICU operations, performance, and practices. For example, one study demonstrated that patients experienced shorter ICU lengths of stay when patient census, number of admissions, and average ICU acuity were higher.^(7)^ Furthermore, when patients were discharged from the ICU during times of higher strain, they had slightly increased odds of ICU readmission.^(7)^

Another study showed that increases in admissions and acuity were associated with shorter times to do-not-resuscitate orders and death within ICUs operating under closed physician staffing models (that is, where all patients are primarily cared for by intensivists),^(8)^ further suggesting that strain impacts patient flow and hence subsequent capacity.

Processes of care also seem to be impacted by strain. For example, one study demonstrated that as admissions and census increase, the odds of appropriate venous thromboembolism prophylaxis decreases, particularly among patients in closed ICUs.^(9)^ Increasing ICU strain also influences physician workflow, with studies showing that strain is associated with increased time spent on direct patient care and trainee education,^(10)^ reduced documentation time,^(10)^ and decreased time spent on newly admitted patients.^(11)^ These studies collectively demonstrate that ICU resources (including clinicians' time) are allocated differently under conditions of strain.

Importantly, these alterations in ICU operations, processes of care, and time allocation do not seem to impact ultimate patient outcomes such as death to as great a degree as might be expected. A large multi-center observational study using ICU census, average patient acuity, and the proportion of new admissions to define ICU capacity strain demonstrated that patients' odds of dying in the hospital were only slightly higher if they were admitted during times of high capacity strain, and that even this small effect was confined to closed ICUs.^(12)^ Similarly, although patients discharged during times of high strain have shorter ICU lengths of stay and more frequent readmissions, they have the same odds of surviving and of returning to home.^(7)^ Thus, it is possible that rather than eroding the quality of care, ICU capacity strain may impact care delivery in ways that make it more efficient, such as by decreasing lengths of stay and shortening time to appropriate decision-making about life support without endangering patients' ultimate outcomes.

## What we don't know about intensive care unit capacity strain

Although the quality and quantity of research surrounding ICU capacity strain has increased dramatically over the past 5 years, there remain substantial gaps in our knowledge. First, the research up to this point has focused on physician workflow, to the exclusion of other disciplines,^(13)^ despite the fact that ICU care is inherently inter-professional. Second, although most prior studies have demonstrated small or no adverse outcomes for patients during times of high strain, there is considerable variability among ICUs, and it seems likely that certain ICUs are more susceptible to adverse effects of strain. Thus, future work is needed to identify heterogeneity in how ICUs that are organized differently respond to strain.

Third, research in different settings is needed to determine whether the effects of strain on processes and outcomes of care exhibit continuous effects across the range of strain, or threshold effects, such that so long as strain is kept below certain definable levels, adverse effects do not manifest. Identifying such "target levels" of strain could help move the field forward to improve the outcomes of critically ill patients. Fourth, rather than examining individual components of strain, such as census and acuity, future studies should seek to develop and validate a composite measure of strain to enhance our understanding of the overall impact of this construct.

Finally, and perhaps most important, the field of strain research has focused primarily on strain within ICUs, with some attention to the impact of ED strain on the outcomes of critically ill patients. Future research needs to assess ward strain and indeed, hospital-wide strain, as hospital units are organizationally dependent and affect each other's capacity and patient flow. Specifically, we need to circle back to apply the knowledge gained from the work in ICU capacity strain to the care of critically ill patients from the moment they step foot into the ED to the time they are discharged from the hospital.

Within the growing body of literature on ICU survivorship, a new line of research needs to focus on hospital wards, the location where the majority of patients are transferred once they have recovered from critical illness. We need to define and operationalize ward capacity strain, in order to determine how it may impact long-term outcomes of patients who are or may become critically ill. Furthermore, future studies should assess the interplay of ward capacity strain with ICU and ED strain and the effects on patient flow, hospital and ICU capacity, and waiting times, in order to better organize patient flow and more efficiently use limited critical care resources.

## Conclusion

ICU capacity strain is associated with physician workflow, processes of care, patient triage, and, in some settings, patient outcomes. Future research should focus on broad ICU populations, on inter-professional clinicians, and on defining and understanding ward strain. Such efforts would improve understanding strain throughout the hospitalizations of critically ill patients, enabling interventions that improve the overall care and outcomes of critically ill patients as well as the efficiency of hospital flow and throughput.
